# Changes in cardiovascular health and white matter integrity with aerobic exercise, cognitive and combined training in physically inactive healthy late-middle-aged adults: the “Projecte Moviment” randomized controlled trial

**DOI:** 10.1007/s00421-023-05319-9

**Published:** 2023-09-28

**Authors:** Francesca Roig-Coll, Alba Castells-Sánchez, Gemma Monté-Rubio, Rosalía Dacosta-Aguayo, Noemí Lamonja-Vicente, Pere Torán-Monserrat, Guillem Pere, Alberto García-Molina, José Maria Tormos, Maria Teresa Alzamora, Dimitriadis Stavros, Marta Sánchez-Ceron, Marc Via, Kirk I. Erickson, Maria Mataró

**Affiliations:** 1 Department of Clinical Psychology and Psychobiology, University of Barcelona, Passeig Vall d’Hebron 171, 08035 Barcelona, Spain; 2 Institut de Neurociències, University of Barcelona, Barcelona, Spain; 3 Medical Psychology Unit, Department of Medicine, University of Barcelona, Barcelona, Catalonia Spain; 4 Centre de Medicina Comparativa i Bioimatge (CMCiB), Institut d’Investigació en Ciències de la Salut Germans Trias I Pujol (IGTP), Badalona, Spain; 5 Unitat de Suport a la Recerca Metropolitana Nord, Fundació Institut Universitari per a la Recerca a l’Atenció Primària de Salut Jordi Gol i Gurina, Mataró, Spain; 6 Institut d’Investigació en Ciències de la Salut Germans Trias i Pujol (IGTP), Badalona, Spain; 7 Institut de Recerca Sant Joan de Déu, Esplugues de Llobregat, Spain; 8 Department of Medicine, Universitat de Girona, Girona, Spain; 9 Institut Guttmann, Institut Universitari de Neurorehabilitació, Universitat Autònoma de Barcelona, Badalona, Spain; 10 Institut de Diagnòstic per la Imatge, Hospital Germans Trias i Pujol, Badalona, Spain; 11 Department of Psychology, University of Pittsburgh, Pittsburgh, USA; 12 AdventHealth Research Institute, Orlando, FL USA; 13 Department of Physical and Sports Education, Faculty of Sport Sciences, PROFITH “PROmoting FITness and Health Through Physical Activity” Research Group, Sport and Health University Research Institute (iMUDS), University of Granada, Granada, Spain

**Keywords:** Aerobic exercise, Computerized cognitive training, Combined training, Cardiovascular health, White matter integrity

## Abstract

**Introduction:**

This is a 12-weeks randomized controlled trial examining the effects of aerobic exercise (AE), computerized cognitive training (CCT) and their combination (COMB). We aim to investigate their impact on cardiovascular health and white matter (WM) integrity and how they contribute to the cognitive benefits.

**Methods:**

109 participants were recruited and 82 (62% female; age = 58.38 ± 5.47) finished the intervention with > 80% adherence. We report changes in cardiovascular risk factors and WM integrity (fractional anisotropy (FA); mean diffusivity (MD)), how they might be related to changes in physical activity, age and sex, and their potential role as mediators in cognitive improvements.

**Results:**

A decrease in BMI (SMD = − 0.32, *p* = 0.039), waist circumference (SMD = − 0.42, *p* = 0.003) and diastolic blood pressure (DBP) (SMD = − 0.42, *p* = 0.006) in the AE group and a decrease in BMI (SMD = − 0.34, *p* = 0.031) and DBP (SMD = − 0.32, *p* = 0.034) in the COMB group compared to the waitlist control group was observed. We also found decreased global MD in the CCT group (SMD = − 0.34; *p* = 0.032) and significant intervention-related changes in FA and MD in the frontal and temporal lobes in the COMB group.

**Conclusions:**

We found changes in anthropometric measures that suggest initial benefits on cardiovascular health after only 12 weeks of AE and changes in WM microstructure in the CCT and COMB groups. These results add evidence of the clinical relevance of lifestyle interventions and the potential benefits when combining them.

**Clinical Trial Registration:**

ClinicalTrials.gov NCT031123900.

## Introduction

In recent decades, there has been a growing interest in identifying the factors that influence cardiovascular, cerebral, and cognitive health during aging, with the aim of delaying or preventing age-related cognitive deficits (Livingston et al. 2017). Literature has identified several modifiable factors and emphasized the link between cardiovascular risk factors, such as hypertension, diabetes, obesity, sedentarism, and smoking, with brain health, specifically for white matter (WM) microstructure (Wassenaar et al. 2019). Accessible, low-cost, and scalable lifestyle interventions, including aerobic exercise (AE) or cognitive training, either individually or in combination, have emerged as promising approaches to improve cardiovascular health (Sisti et al. 2018; Zhang et al. 2017) and protect brain (Wassenaar et al. 2019) and cognition (Phillips 2017; Sprague et al. 2019).

Physical activity (PA) has been widely recognized for its numerous beneficial effects on cardiovascular health (Fontana 2018; Nystoriak and Bhatnagar 2018; Pinckard et al. 2019). Regular PA improves glucose tolerance, insulin sensitivity, and reduces circulating lipid concentrations, resting heart rate, and blood pressure (Nystoriak and Bhatnagar 2018; Pinckard et al. 2019). Physically active individuals tend to have higher levels of high-density lipoprotein (HDL) (Kodama et al. 2007), and higher physical fitness is associated with lower blood pressure in cross-sectional and longitudinal studies (Bacon et al. 2004). A meta-analysis by Lin et al. (2015) showed that exercise interventions increase cardiorespiratory fitness (CRF) and improve lipid profiles, including lower triglyceride levels and higher HDL levels. The specific outcomes of AE trials, however, are confounded by the heterogeny of factors such as age, sex, and health status of participants, as well as the type of exercise program used (Kodama et al. 2007; Lin et al. 2015). In the context of brain health, there has been a growing interest in examining the relationship between PA or AE and the health of white matter (WM). However, the findings from various studies have been inconsistent, yielding diverse results (Sexton et al. 2016). Some studies have reported positive correlations between greater PA engagement and increased WM volumes, improved WM microstructure, and lower volume and intensity of WM lesions (Sexton et al. 2016). Conversely, other studies have presented negative results (Burzynska et al. 2014; Marks et al. 2011; Tian et al. 2014a, b). Regarding WM microstructure, cross-sectional studies have suggested that higher levels of PA are associated with increased global and local fractional anisotropy (FA) in the corpus callosum, superior longitudinal fasciculus, and arcuate fasciculus (Gow et al. 2012; Johnson et al. 2012; Liu et al. 2012). However, a randomized controlled trial (RCT) involving healthy sedentary older adults did not find significant changes in WM integrity following a 1-year AE intervention, despite improvements in aerobic fitness related to increased WM integrity in frontal and temporal lobes (Voss et al. 2013). Similarly, more recent RCTs investigating the effects of AE programs, lasting 3 sessions per week for 3 or 6 months in healthy older adults, did not yield significant results related to WM microstructure (Clark et al. 2019; Sexton et al. 2020). These inconclusive findings may be influenced by the heterogeneity of exercise program parameters (FITT-VP: Frequency, Intensity, Time, Type, Volume, and Progression) and individual characteristics, such as sex and genetics, which can influence the impact of AE on WM in late-life (Stillman et al. 2020). Based on the findings from several systematic reviews, AE interventions have demonstrated positive effects on executive function, attention, and speed, with a small-to-moderate effect size (Barha et al. 2017; Colcombe and Kramer 2003; Northey et al. 2018; Stillman et al. 2020). Understanding the underlying cardiovascular factors influencing these outcomes is of utmost importance.

While cognitive training (CT) has shown effectiveness in improving cognitive performance within trained domains for older adults, its transfer to non-trained domains is limited (Sprague et al. 2019; Wassenaar et al. 2019). Existing evidence has linked cardiovascular risk factors to cognitive performance and risk of dementia (Baumgart et al. 2015; Iadecola 2014; Qiu et al. 2005), but direct effects of CT on these variables remain lacking. Nevertheless, CT has shown effects on the brain structure and function of healthy older adults (Belleville and Bherer 2012; McPhee et al. 2019), with recent interventional studies suggesting that both single and multi-domain CT interventions can improve WM integrity (Wassenaar et al. 2019). In single-domain CT trials assessing memory training's effect on WM microstructure in older adults, control groups showed widespread deterioration (decreased FA and increased MD), while training groups showed no such decline after 8 and 10 weeks of intervention (Engvig et al. 2012; de Lange et al. 2016, 2017). Conversely, findings from 12-week multi-domain CT programs have been inconsistent compared to control groups (Chapman et al. 2015; Cao et al. 2016; Lampit et al. 2015). In a longer 6-month multi-domain CT intervention, improvements in WM microstructure (increased FA and decreased MD) were observed in the corpus callosum genus of older adults but not in the control group (Lövdén et al. 2010). Although heterogeneity of these results has been related to the FITT-VP parameters of the cognitive programs and sample characteristics of these trials, authors highlight the potential benefits of CT in the frontal and medial brain regions for mitigating age-related WM microstructure decline (McPhee et al. 2019).

Recent evidence suggests that the combination (COMB) of AE and CT may have complementary and additive effects on cognition and brain health (Joubert and Chainay 2018; Ten Brinke et al. 2020). This hypothesis led to increased interest on the mechanisms involved in the potential COMB-related benefits such as growth factors and inflammatory profiles (Anderson-Hanley et al. 2012; Castells-Sánchez et al. 2022; Rahe et al. 2015). While AE interventions have shown positive effects on lipid profiles and blood pressure (Fagard 2001; Kodama et al. 2007; Lin et al. 2015), suggesting that a combined AE and CT intervention (COMB) could yield similar results, to our knowledge no studies have explored the impact of COMB-related changes on cardiovascular variables. Additionally, there is limited evidence regarding the effects of COMB training on WM microstructure. Lövdén et al. (2012) observed a trend for decreased MD in the right hippocampus in healthy older men after 4 months of COMB training, whereas those in the walking program only did not show significant changes. Similarly, Takeuchi et al. (2020) reported a significant decrease in MD in multiple frontal and subcortical brain areas after 12 weeks of COMB training in healthy older adults compared to those in single working memory or AE groups. Further research is needed to fully understand the benefits of COMB training, its underlying mechanisms, and the potential moderating effects of variables such as age, sex, and genetics (Joubert & Chainay 2018).

“Projecte Moviment” is a randomized controlled trial (RCT) investigating the impact of a high-frequency (5 days per week), short-term (12 weeks) program involving aerobic exercise (AE), computerized cognitive training (CCT), and their combination in healthy, physically inactive older adults (Castells-Sánchez et al. 2019). The findings on cognition, psychological status, physical activity, molecular biomarkers, and brain volume outcomes have been previously published in Roig-Coll et al. (2020) and Castells-Sánchez et al. (2022). Our study offers a unique opportunity to compare and disentangle the effects of physical activity and cognitive training within a unified framework, exploring the potential additive effects of their combined intervention on cardiovascular risk factors and brain structure and functions. Moreover, we aim to investigate the underlying mechanisms and mediating factors, enabling the personalization of interventions to maximize their benefits.

## Methods

### Study design

“Projecte Moviment” is a multi-center, single-blind RCT developed between November 2015 and April 2018 by the University of Barcelona in collaboration with Institut Universitari d’Investigació en Atenció Primària Jordi Gol, Hospital Germans Trias i Pujol and Institut Guttmann. Participants were assigned to four parallel groups: an AE group, a CCT group, a COMB group and a waitlist control group. Interventions lasted 12 weeks, and the assessments were conducted at baseline and trial completion. This research project was approved by the responsible ethics committees (Bioethics Commission of the University of Barcelona -IRB00003099- and Clinical Research Ethics Committee of IDIAP Jordi Gol -P16/181-) following the Declaration of Helsinki and was registered in ClinicalTrials.gov (NCT031123900). This research paper follows the previously published protocol (Castells-Sánchez et al. 2019) and complements previously published results (Castell-Sánchez et al. 2022; Roig-Coll et al. 2020).

### Participants

Healthy adults aged 50–70 years were recruited using lists of patients of general physicians and volunteers from previous studies, as well as via advertisements and oral presentations in health care centers, local community centers and the local media in the Barcelona metropolitan area. Sample size estimation considered previous studies (Colcombe and Kramer 2003; Erickson et al. 2011;) and was performed using PASS 14 Power Analysis and Sample Size Software (Castells-Sánchez et al. 2019). Participants were informed and screened with a phone call and on-site interview. If they met the inclusion and exclusion criteria (see Table 1), they were selected and gave written informed consent prior to study commencement. After the baseline assessment, participants were randomly assigned to four parallel groups, an AE group performing physical activity, a CCT group using the Guttmann Neuropersonal Trainer online platform (GNPT®, Spain; Solana et al. 2014, 2015), a COMB group combining both training programs, and a waitlist control group. The allocation sequence was designed by a statistician and consisted of a random combination of sex, age and years of education, allowing for balanced group allocations accounting for these demographics. The intervention team was aware of the allocation, but the assessors remained blind. Extended details are included in Castells-Sánchez et al. (2019).

**Table 1:** Inclusion and exclusion criteria for Projecte Moviment

Inclusion criteria	Exclusion criteria
Aged 50–70 years	Current participation in any cognitive training activity or during last 6 months > 2 h/week
≤ 120 min/week of physical activity during last 6 months	Diagnostic of dementia or mild cognitive impairment
Mini-mental state examination (MMSE) ≥ 24	Diagnostic of neurological disorder: stroke, epilepsy, multiple sclerosis, traumatic brain injury, brain tumor
Montreal cognitive assessment 5-min (MoCA 5-min) ≥ 6	Diagnostic of psychiatric illness current or during last 5 years
Competency in Catalan or Spanish	Geriatric depression scale (GDS-15) > 9
Adequate visual, auditory and fine motor skills	Consumption of psychopharmacological drugs current or during last 5 years; or more than 5 years throughout life
Acceptance of participation in the study and signature of the informed consent	History of drug abuse or alcoholism current or during last 5 years; or more than 5 years throughout life; > 28 men and > 18 women unit of alcohol/week
	History of chemotherapy
	Contraindication to magnetic resonance imaging

MMSE (Blesa et al. 2001); MoCA 5-min (Wong et al. 2015); GDS-15 (Martínez et al. 2002)

### Interventions

Interventions were home-based, scheduled five days per week for 12 weeks and applied as individual programs. Participants were monitored during the intervention: they received phone calls every two weeks, a mid-point visit after six weeks of the intervention, and a final visit. The participants were asked about their level of compliance, interfering events, satisfaction, motivation, and difficulty level. They also registered the training frequency and adverse events occurring during the intervention in a follow-up diary. The AE and the COMB group were asked to record the intensity at which they performed the exercise based on BRPES values the Borg Rating of Perceived Exertion Scale (BRPES; Borg 1982). The BRPES offers a broad range of values and exertion labels, making it easier to differentiate between perceived effort levels, especially between "very light" (9–10) and "somewhat hard" (13–14). This is particularly useful for our sedentary (non-sportive) sample unfamiliar with exercise-related perception and vocabulary. Participants were trained to monitor their activity in a diary, registering the activity's date and duration, any adverse events occurring, and the intensity of the walking using BRPES values. CCT compliance was registered in the GNPT online platform. We ensured that all sources of compliance information were coherent and allowed us to obtain the level of adherence.

Participants allocated to the AE group followed a progressive brisk walking program. They started (week 1) walking 30 min per day at BRPES 9–10 intensity; the following week (week 2), the duration was increased to 45 min per day; during the remaining ten weeks, they had to walk 45 min per day at BRPES 12–14. Participants allocated to the CCT group performed 45 min sessions of multi-domain computerized cognitive training using the GNPT software platform. Cognitive tasks targeted executive function, visual and verbal memory and sustained, divided and selective attention. The GNPT platform adjusted the task demand for each participant based on their baseline cognitive profile and the scores of the activities. Participants allocated to the COMB group conducted the brisk walking program and the CCT following the same instructions. They had to perform AE and CCT separately, in a single continuous bout of 45 min for each intervention and without any order or timepoint restriction. Participants allocated to the waitlist control group were on the waiting list for 12 weeks and were asked not to change their regular lifestyle. After the RCT, they were offered to enroll in one of the treatments, but their data after the intervention were not included in the analysis. The protocol for each intervention condition is explained in more detail in Castells-Sánchez et al. (2019).

### Assessment/outcomes

#### Cardiovascular risk factors

Demographic characteristics and medical history were collected by nurses in the Primary Health Care Centers. They registered cardiovascular health variables, including history and treatment of diabetes, hypertension, dyslipidemia and current smoking status.

##### Anthropometric and cardiovascular measures

Weight and height were measured using standardized anthropometric procedures without shoes in an upright standing position. Body mass index (BMI) was calculated as weight (kg) divided by the square of height (m^2^). Waist circumference (cm) was measured at the mid-point between the bottom of the rib cage and the iliac crest. After resting for 5 min, heart rate (beats/min) and systolic and diastolic blood pressures (mmHg) were measured using an automated machine (Omron M2 Basic). Participants sat comfortably with their arms resting on the table at heart level. Two measurements were taken for at least 1 min, and their average was used.

##### Blood sample biomarkers

Following an overnight fast, phlebotomy was conducted between 8:00 and 9:30 to determine the hemogram and lipid profiles. Blood samples were obtained from the antecubital vein and collected in EDTA tubes for plasma analyses. Tubes were immediately transferred to the Dr Robert Primary Health Center and Laboratori Clínic Metropolitana Nord, Germans Tries i Pujol, Gerència Territorial Metropolitana Nord, Institut Català de la Salut, where the samples were processed upon arrival following standard operating procedures. Serum concentrations of glucose (mg/dL), triglycerides (mg/dL), total cholesterol (mg/dL), HDL (mg/dL) and low-density lipoprotein (LDL) (mg/dL) were determined and selected for this paper.

#### Neuroimaging: DTI acquisition and preprocessing

MRI data were collected at the Hospital Germans Trias i Pujol using a 3 T Siemens Magnetom Verio Symo MR B17 (Siemens 243 Healthineers, Erlangen, Germany). The scanning protocol included high-resolution 3-dimensional T1-weighted images acquired in the transverse plane (TR = 1900 ms, TE = 2.73 ms, 192 slices, FOV = 230 mm; 0.9 × 0.9 × 0.9 mm isotropic voxel). DTI images were acquired in the transverse plane, AP phase encoding direction (TR = 10,200 ms, TE = 89 ms, FOV = 230 mm; 2.0 × 2.0x2.0 mm isotropic voxel; number of directions = 64, b-value = 1000 s/mm^2^, b_0_ value = 0 s/mm^2^). DTI and T1 images were visually inspected for artefacts. From an initial sample of 82 participants, images of 12 subjects were not acquired for personal or technical issues, and 20 were excluded for movement artefacts in the DTI.

First, data were eddy-corrected, and the diffusion gradient vectors (bvecs) were rotated accordingly using FDT (FMRIB’s Diffusion Toolbox), part of FSL (FMRIB's Software Library) (Behrens et al 2003, 2007). Moreover, Bias Field Correction (BFC) was estimated, and DTI data were corrected using the Brain Suite Package (http://brainsuite.org/) to correct the EPI distortions. Next, the FDT’s DTIFIT function was applied to the corrected DTI images to fit a diffusion tensor model at each voxel to obtain the FA and MD images.

We used TBSS, part of FSL (Tract-Based Spatial Statistics; Smith et al. 2006), to perform statistical analyses on the FA and MD images. TBSS performed non-linear registration (using FNIRT) of the FA images to the MNI standard space and generated a mean FA skeleton representing the center of the WM tracts common to the whole sample. Each subject’s FA image was projected onto the skeleton to obtain the individual FA skeleton images registered in the common space. Only tracts with an average FA ≥ 0.2 across the sample were considered. In addition, FA maps representing the pre-to-post changes were created for each individual. The same steps were applied to the MD maps. Finally, pre-test and post-test global mean estimates of FA and MD were also extracted and exported to SPSS for statistical analyses.

#### Physical activity

Physical activity levels were assessed with the Spanish-validated short version of the Minnesota Leisure Time Physical Activity Questionnaire (MLTPAQ; Ruiz Comellas et al. 2012). Participants reported the frequencies and durations of several activities—sportive walking, sport/dancing, gardening, climbing stairs, shopping walking and cleaning house—during the last month. We obtained energy expenditure for each activity by transforming monthly hours of activities into units of Metabolic Equivalent of Tasks (METs). We calculated the METs spent in Sportive Physical Activity (S-PA) by adding the categories of sportive walking and sport/dancing.

#### Cardiorespiratory fitness

CRF was evaluated by conducting the Rockport 1-Mile Test, which consisted of walking one mile on a treadmill while adjusting their speed to be as fast as possible without running. We registered average speed during the test, time to complete the mile, and heart rate at the end of the test. Maximal aerobic capacity (VO_2max_) was estimated with the standard equation developed by Kline et al. (1987) and using the following variables: weight, age, sex, time to complete the mile, and heart rate at the end of the test.

#### Cognitive performance

An extensive neuropsychological battery was administered in a single session of 60–90 min in the same order for all participants and before the CRF test or any type of exercise to exclude the acute effect of the exercise on cognitive performance. Tests included in the battery obtained measure of multiple cognitive functions grouped by a theoretically-driven approach (Strauss and Spreen 1998; Lezak et al. 2012): Flexibility (Trail Making Test B-A time; Tombaugh 2004), Fluency (letter and category fluency; Peña-Casanova et al. 2009), Inhibition (interference-Stroop Test; Golden 2001), Working Memory (backward-WAIS-III; Wechsler, 2001), Visuospatial Function (copy accuracy-Rey Osterrieth Complex Figure; Rey 2009), Language (Boston Naming Test-15; Goodglass et al. 2001), Attention (forward span, digit symbol coding and symbol search WAIS-III; Wechsler, 2001), Speed (Trail Making Test-A; Tombaugh 2004; copy time-Rey Osterrieth Complex Figure; Rey 2009), Visual Memory (memory accuracy-Rey Osterrieth Complex Figure; Rey 2009) and Verbal Memory (total learning and recall-II Rey Auditory Verbal Learning Test; Schmidt 1996). The measures were transformed into six general domains: (1) executive function, (2) visuospatial function, (3) language, (4) attention-speed, (5) memory and (6) global cognitive function. Extended details can be found in Supplementary Table 1.

### Statistical analyses

Statistical procedures were performed using IBM SPSS Statistics 24. First, the distribution of raw scores was assessed for normal distribution (i.e., outliers, skewness). Then, we calculated pre-to-post change scores, compared baseline scores between groups and performed cross-time partial correlations to detect potential confounds and ceiling effects.

The intervention effect (i.e., change between baseline and follow-up) on cardiovascular risk factor variables and global FA and MD were examined for each group using paired-sample t-tests. In order to test the specificity of the effects, we performed linear regression models using a dummy codification for the *treatment* variable (AE vs control, CCT vs control and COMB vs control). The models included changes in each cardiovascular risk factor variable and the global FA and MD as dependent variables and the baseline outcome score, sex, age, years of education and the treatment variables (AE vs control, CCT vs control and COMB vs control) as independent variables. In the models with cardiovascular risk factors, we also adjusted for BMI, current smoking status and use of dyslipidemia, diabetes and hypertension medication.

We employed partial correlation to assess whether the previously reported significant intervention-related changes in S-PA and CRF observed in the AE and COMB groups (Roig-Coll et al. (2020) were related to cardiovascular risk factors and global FA and MD changes. The analysis was controlled for sex, age, years of education. We also controlled for BMI in cardiovascular risk factor correlations.

We used the PROCESS Macro for SPSS (Hayes 2017) to analyze the moderating effect of age and sex on intervention-related cardiovascular risk factors and global FA and MD changes. We also used the PROCESS macro to perform mediation analyses to assess whether these changes mediated the cognitive benefits observed in the AE and COMB groups (Roig-Coll et al. 2020). For mediation analyses, we introduce the treatment variable (condition vs control) as the independent variable, changes in cognitive functions with significant intervention-related changes as the dependent variables and cardiovascular risk factors and in global FA and MD changes as mediators while controlling for baseline performance score, age, sex and years of education (BMI was only introduced in the cardiovascular risk factor models). These analyses considered the bias-corrected 95% confidence intervals (CIs) based on 5000 bootstrapped samples, and the significance of mediation was indicated if the CIs did not overlap with 0 (Hayes 2017).

### Neuroimage analyses

The tract-wise non-parametric inference was computed using the FSL’s randomise tool (Winkler et al. 2014) on the preprocessed FA and MD images based on 5000 permutations. The following models were considered for FA and MD maps separately: (1) One-way ANOVA including the baseline maps for the four groups (AE, CCT, COMB and control) to assess potential baseline differences; (2) within-group paired t-test to assess pre-to-post changes for each group (AE, CCT, COMB and control); (3) two-sample t-test comparing pre-to-post changes in FA and MD in each intervention group compared to that in the control group (i.e., AE vs control; CCT vs control; COMB vs control); (4) partial correlations between changes in the FA and MD and changes in CRF and S-PA in the AE and COMB groups. Age, sex and years of education were used as covariates in all models. Statistical significance < 0.05 was accepted. For multiple comparisons across space, we used the family-wise error rate (FWE) correction.

## Results

### Participants

**Fig. 1:**
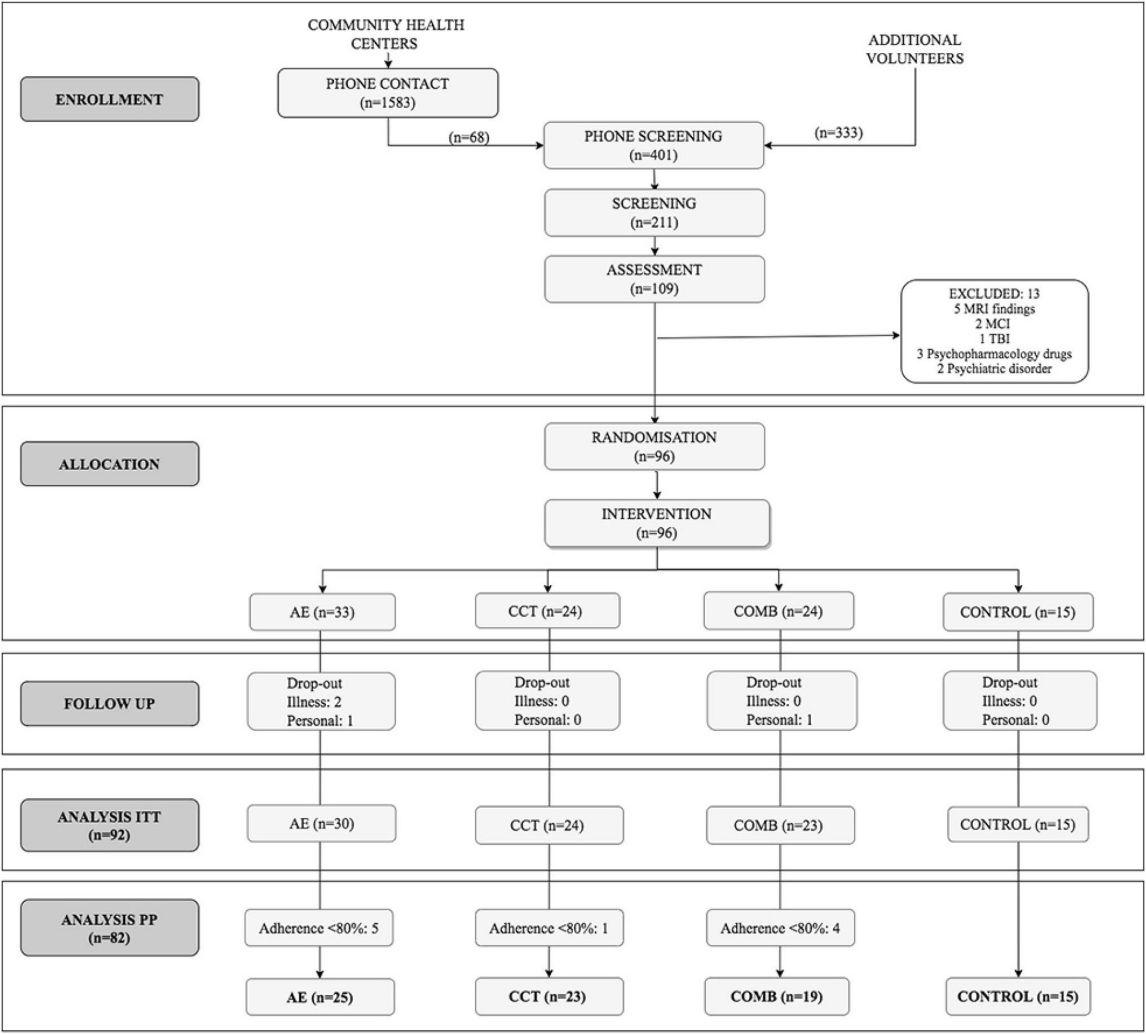
Flow chart

A total of 109 participants completed the baseline assessment and 92 completed the intervention (intention to treat sample, ITT) (see Fig. 1). As we published in the “Projecte Moviment” Protocol (Castells-Sánchez et al. 2019), we conducted analyses in the Per Protocol (PP) sample which included 82 participants (62% female; age = 58.38 ± 5.47) with a level of adherence > 80% (see Table 2). There were no significant differences in demographic variables between the groups of the ITT sample and between the ITT and the PP samples (see Supplementary Tables 2 and 3).

**Table 2:** Participants characteristics at baseline

	Total Mean (SD)	AE Mean (SD)	CCT Mean (SD)	COMB Mean (SD)	Control Mean (SD)	Group comparison
n total/n females	82/51	25/13	23/16	19/14	15/8	*Χ*^*2*^(3) = 3.20, * p* = 0.361
Age (years)	58.38 (5.47)	58.40 (5.12)	57.91 (5.31)	60.32 (5.54)	56.60 (5.97)	H(3) = 3.53, * p* = 0.317
Years of education	12.52 (5.57)	12.44 (5.75)	12.04 (4.94)	12.37 (5.43)	13.60 (6.72)	H(3) = 0.28, * p* = 0.963
Vocabulary subtest (WAIS-III)	44.14 (8.30)	43.92 (9.53)	44.26 (7.16)	44.53 (8.02)	43.80 (8.98)	F(3,77) = 0.03, * p* = 0.993
BMI (kg/m^2^)	28.63 (4.96)	28.14 (5.53)	28.13 (4.26)	28.37 (4.42)	30.49 (5.70)	H(3) = 1.72, * p* = 0.632
Diabetes (n)	10	2	2	1	5	*Χ*^*2*^(3) = 7.79, * p* = 0.051
Hypertension (n)	17	3	6	4	4	*Χ*^*2*^(3) = 1.88, *p* = 0.597
Dyslipidemia (n)	30	8	8	7	7	*Χ*^*2*^(3) = 0.92, *p* = 0.821
Current smokers (n)	9	3	6	0	0	*Χ*^*2*^(3) = 9.59, *p* = 0.022*
Use of cholesterol, diabetes and/or hypertension medication (n)	22	6	6	5	5	*Χ*^*2*^(3) = 0.43, *p* = 0.933

*AE* aerobic exercise, *BMI* body max index, *CCT* computerized cognitive training, *COMB* combined training, *WAIS-III* Wechsler adult intelligence scale

Mean (SD); X^2^ = chi square; H = Kruskall Wallis H test; F = Anova test

See Supplementary Tables 4 for more cognitive, physical, cardiovascular risk factors, global FA and MD outcomes at Baseline

The PP sample showed no significant baseline between-group differences in demographic, cardiovascular risk factors, global FA and MD, physical and cognitive scores, except for Non-Sportive PA and current smoking status (see Table 2 and Supplementary Tables 4 for extended details). Therefore, current smoking status at baseline was included as a covariate in further analyses. Whole brain voxel-wise analysis did not show significant between-group differences in diffusivity measures at baseline.

### Intervention-related changes in cardiovascular risk factors

Within-group analyses showed significant changes between baseline and follow-up. We found significant decrease in BMI (*t*(22) = 2.12; *p* = 0.046), HDL (*t*(22) = 2.33; *p* = 0.029), waist circumference (*t*(24) = 4.13; *p* < 0.001) and diastolic blood pressure (*t*(24) = 2.46; *p* = 0.022) for the AE group, significant decrease in triglycerides (*t*(20) = 2.14; *p* = 0.045) and systolic blood pressure (*t*(22) = 2.40; *p* = 0.026) for the CCT group, and significant decrease in BMI (*t*(18) = 2.11; *p* = 0.049) and total cholesterol (*t*(18) = 2.24; *p* = 0.038) for the COMB group. There was also a significant increase in diastolic blood pressure (*t*(14) = -2.29; *p* = 0.038) for the waitlist control group (see Supplementary Table 5).

Contrasts between each intervention and waitlist control group for cardiovascular risk factors outcomes are reported in Table 3. Compared to the waitlist control group, we found significant AE-related changes in BMI, waist circumference and diastolic blood pressure, significant CCT-related changes in triglycerides and diastolic blood pressure, and significant COMB-related changes in BMI and diastolic blood pressure.

**Table 3:** Comparison of intervention-related changes (vs. the control group) in cardiovascular risk factors for each intervention group

	AE vs control B (95% CI), SMD, *p* value	CCT vs control B (95% CI), SMD, *p* value	COMB vs control B (95% CI), SMD, *p* value
BMI (kg/m^2^)	− 0.51, (− 0.99, − 0.03), SMD = − 0.32, *p* = 0.039*	− 0.24 (− 0.73, 0.25), SMD = − 0.15,* p* = 0.327	− 0.57, (− 1.08, − 0.05), SMD = − 0.34, *p* = 0.031*
Glucose (mg/dL)	− 5.70, (− 22.48, 11.08), SMD = − 0.11, *p* = 0.500	− 11.20, (− 28.60, 6.20), SMD = − 0.21, *p* = 0.203	− 6.10, (− 23.82, 11.62), SMD = − 0.11, *p* = 0.494
Triglycerides (mg/dL)	− 13.44, (− 34.99, 8.12), SMD = − 0.13, *p* = 0.218	− 24.40, (− 46.69, − 2.11), SMD = − 0.23, *p* = 0.032*	− 19.43, (− 42.91, 4.04), SMD = − 0.18, *p* = 0.103
Total cholesterol (mg/dL)	2.07, (− 13.16, 17.29), SMD = 0.04, *p* = 0.787	− 2.93, (− 18.66, 12.80), SMD = − 0.06, *p* = 0.711	− 8.92, (− 25.26, 7.40), SMD = − 0.17, *p* = 0.279
HDL (mg/dL)	0.33, (− 2.89, 3.55), SMD = 0.03, *p* = 0.837	0.34, (− 2.90, 3.58), SMD = 0.03, *p* = 0.834	1.59, (− 1.87, 5.05), SMD = 0.15, *p* = 0.363
LDL (mg/dL)	− 2.60, (− 17.23, 12.03), SMD = − 0.06, *p* = 0.724	0.41, (− 14.43, 15.26), SMD = 0.01, *p* = 0.956	− 8.54, (− 24.14, 7.06), SMD = − 0.17, *p* = 0.278
Waist circumference (cm)	− 4.90, (− 8.10, − 1.70), SMD = − 0.42, *p* = 0.003**	− 1.21, (− 4.56, 2.15), SMD = − 0.10, *p* = 0.474	− 1.50, (− 5.07, 2.07), SMD = − 0.12, *p* = 0.404
Systolic blood pressure (mmHg)	− 5.42, (− 13.69, 2.86), SMD = − 0.20, *p* = 0.196	− 6.40, (− 14.79, 2.00), SMD = − 0.24, *p* = 0.133	− 5.61, (− 14.50, 3.29), SMD = − 0.19, *p* = 0.213
Diastolic blood pressure (mmHg)	− 7.55, (− 12.84, − 2.26), SMD = − 0.42, *p* = 0.006**	− 6.30, (− 11.64, − 0.95), SMD = − 0.35, *p* = 0.022*	− 6.15, (− 11.81, − 0.49), SMD = − 0.32, *p* = 0.034*
Resting heart rate (beats/min)	1.61, (− 2.98, 6.21), SMD = 0.09, *p* = 0.485	− 0.16, (− 4.87, 4.56), SMD = − 0.01, *p* = 0.947	0.69, (− 4.15, 5.53), SMD = − 0.04, *p* = 0.775

Covariates: sex, age, years of education, BMI, baseline, smoking status, use of cholesterol, diabetes and/or hypertension medication

*AE* aerobic exercise, *BMI* body mass index, *CCT* computerized cognitive training, *COMB* combined training, *HDL* high density lipoprotein, *LDL* low density lipoprotein

SMD = β

*p < 0.05, **p < 0.01

### Intervention-related changes in WM integrity

**Fig. 2:**
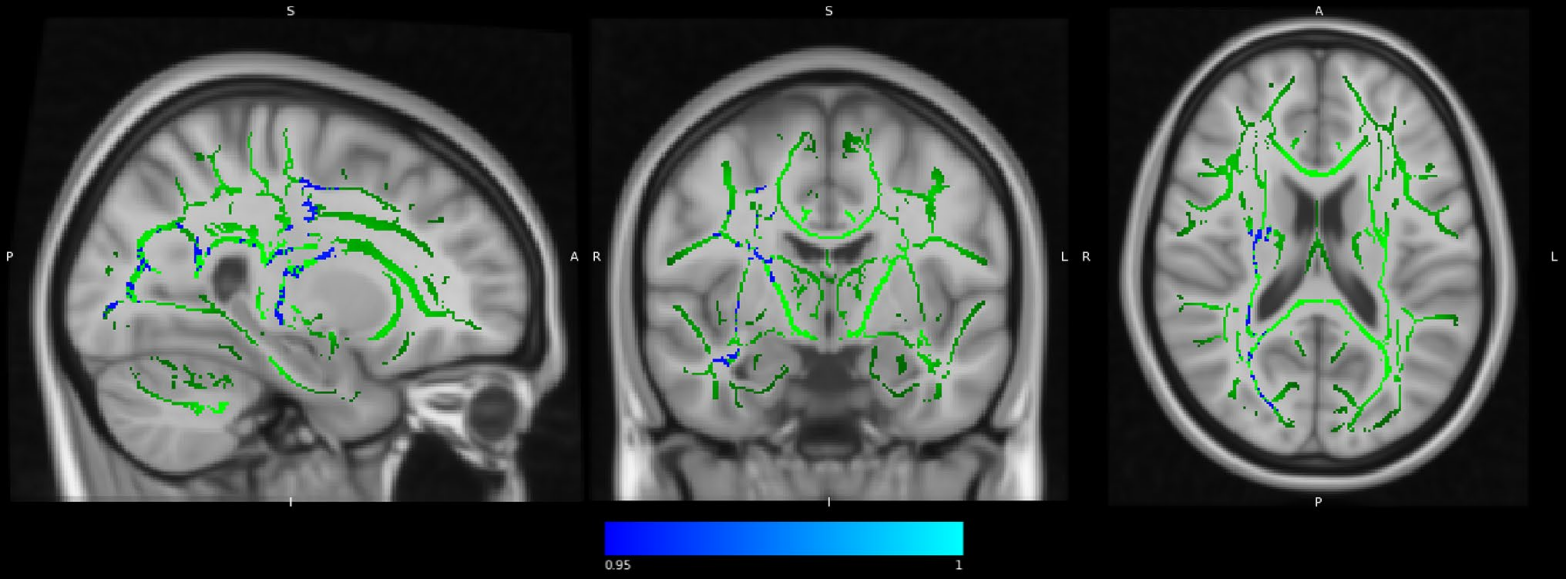
Significant changes in MD map in COMB group between baseline and follow-up (i.e., effect of time). In blue-light blue clusters with significant decrease of MD (MNI coordinates: 26, − 7, 18). Statistical maps are represented in radiological convention superimposed on an MNI152 template. The threshold for significance was set at p < 0.05 corrected for multiple comparisons across space using family-wise error rate (FWE)

There were no significant changes in global FA and MD between baseline and follow-up for any of the AE, CCT, COMB and waitlist control groups, while there was a tendency of reduced global MD (t(13) = 2.020; *p* = 0.064) in the COMB group (Supplementary Table 6). TBSS analysis revealed significant clusters within the WM skeleton with decreased MD when comparing the baseline with the follow-up maps in the COMB group in several areas, including the right sub-global extranuclear WM, right precentral gyrus, and right cingulate gyrus (see Fig. 2 and Table 4). TBSS analysis did not show any significant changes in FA and MD between baseline and follow-up in AE, CCT and waitlist control groups.

**Table 4:** Intra-group intervention related changes in WM integrity in COMB group: clusters with significant MD changes between baseline and follow-up

Anatomic label Tailarach (JHU tracts)	Coordinates MNI x, y, z (mm)	MAX	Cluster size
MD			
Sub-global extranuclear WM R	26, − 7, 18	0.958	3297
Frontal lobe, precentral gyrus WM R (32% superior longitudinal fasciculus)	43, − 9, 26	0.951	46
Limbic lobe, cingulate gyrus WM R	15, − 37, 37	0.95	23

The threshold for significance was set at *p* < 0.05 corrected for multiple comparisons across space using family-wise error rate (FWE). Extent threshold cluster size < 10

*COMB* combined training, *MD* mean diffusivity, *R* right, *WM* white matter

**Fig. 3:**
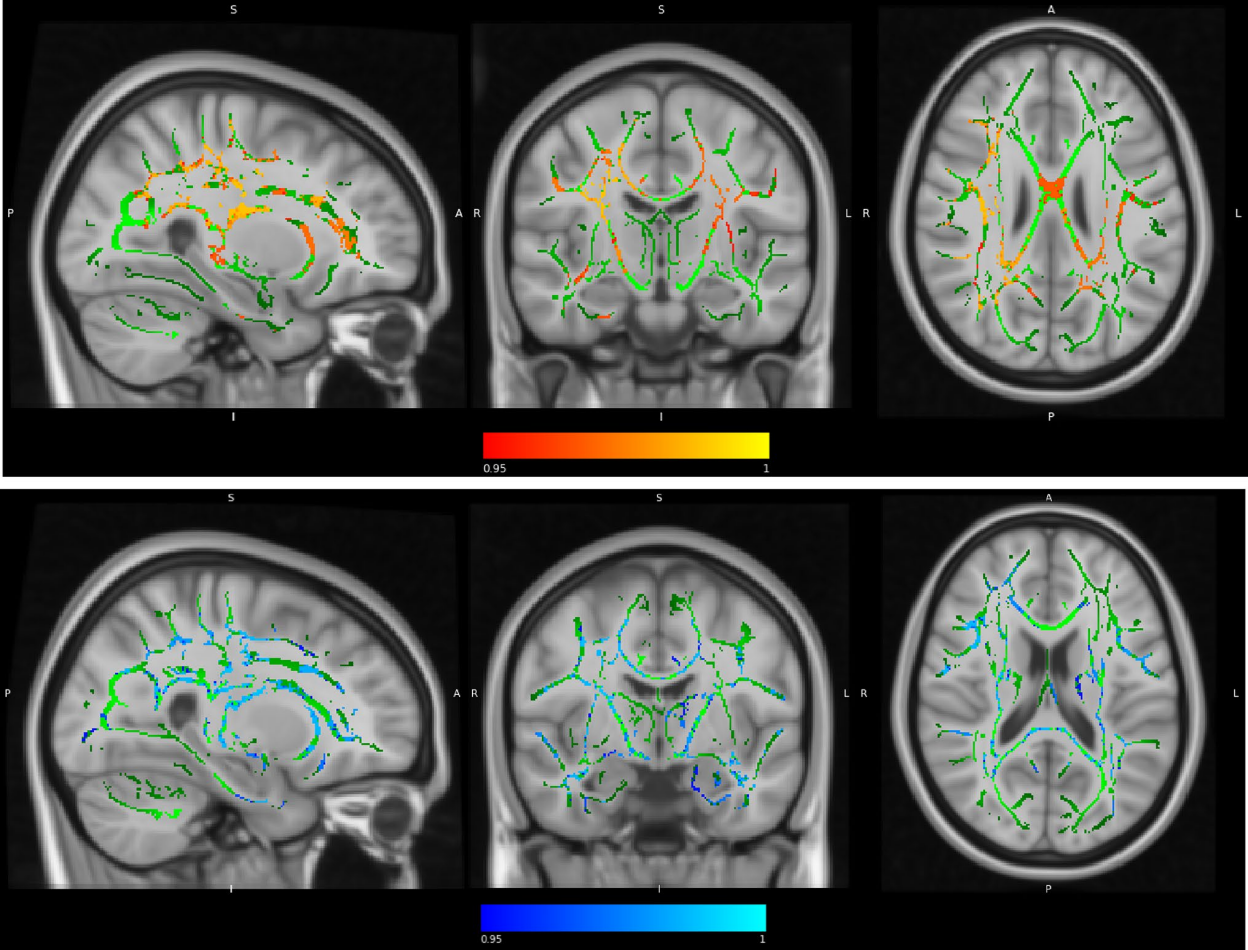
Significant changes in FA ad MD in COMB group compared to the waitlist control group (i.e., interaction between the effects of time and group allocation). In red-yellow, clusters with significant increase in FA (MNI coordinates: 28, − 14, 26). In blue-light blue, clusters with significant decrease of MD (MNI coordinates: 26, − 7, 18). Statistical maps are represented in radiological convention superimposed on an MNI152 template. The threshold for significance was set at p < 0.05 corrected for multiple comparisons across space using family-wise error rate (FWE)

Results of linear regression models which tested significant changes in global FA and MD between each intervention group compared to control showed a significant reduction in MD only in CCT compared to control (SMD = − 0.34; *p* = 0.032) (See Supplementary Table 7 for extended details). TBSS analysis reported significant intervention-related changes in the FA and the MD only in the COMB group compared to waitlist controls in several areas. The main FA significant clusters correspond to the right subgyral of the frontal lobe, other minor clusters placed in the left subgyral and globus pallidus of the temporal lobe (see Fig. 3 and Table 5). The only MD significant cluster correspond to right sub-global extranuclear WM.

**Table 5:** Intervention related changes in WM integrity: clusters with significant FA and MD changes between COMB vs control groups

Anatomic label Tailarach (JHU tracts)	Coordinates MNI x, y, z (mm)	MAX	Cluster size
FA			
Frontal lobe, subgyral WM R (corticoespinal tract 18%)	28, − 14, 26	0.989	21,467
Left brainstem, midbrain	− 18, − 30, − 2	0.951	61
Temporal lobe, subgyral WM L (uncinate fasciculus 8%)	− 37, − 8, − 20	0.95	20
Temporal lobe, subgyral WM L (inferior longitudinal fasciculus 13%)	− 41, − 10, − 25	0.95	18
Lateral globus pallidus WM L	− 27, − 18, − 8	0.95	14
Temporal lobe, subgyral WM L (inferior longitudinal fasciculus 13%)	− 41, − 10, − 25	0.95	10
MD			
Sub-global extranuclear WM R	26, − 7, 18	0.989	35,827

*COMB* combined training, *FA* fractional anisotropy, *L* left, *MD* mean diffusivity, *R* right, *WM* white matter

The threshold for significance was set at *p* < 0.05 corrected for multiple comparisons across space using family-wise error rate (FWE). Extent threshold cluster size < 10

### Relation between cardiovascular risk factors and WM integrity with physical activity outcomes

Our previous study reported significant intervention-related changes in S-PA and CRF levels in the AE and COMB groups (Roig-Coll et al. 2020). Our current study found no significant relationship between the increased S-PA and CRF levels and the changes in cardiovascular risk factors and the global FA and MD for the AE and COMB groups. TBSS analysis did not resulted in any significant correlation between increased CRF and S-PA levels and the FA and MD changes.

### The moderation effects of sex and age

Moderation analyses showed that age did not significantly moderate the effects of the intervention on the cardiovascular risk factors and the global FA and MD in any group. In the AE group, sex (women = 1, men = 0) moderated the effects of intervention on systolic pressure (β = − 16.47, t = − 2.69, *p* = 0.009), LDL levels (β = 28.22, t = 2.47, *p* = 0.016) and waist circumference (β = − 5.81, t = − 2.44, *p* = 0.017). In the COMB group, sex also moderated the effects of the intervention on systolic (β = 15.76, t = 2.23, *p* = 0.029) and diastolic pressure (β = 9.67, t = 2.13, *p* = 0.037). In the CCT group, sex also moderated the change in waist circumference (β = 6.06, t = 2.49, *p* = 0.012). Sex did not significantly moderate the effects of the intervention on global FA and MD in any group.

### Mediation effects on intervention-related cognitive benefits

We applied mediation analyses to examine whether changes in cardiovascular risk factors and global FA and MD mediated the relationship between the intervention and the improvement in cognitive domains as reported in Roig-Coll et al. (2020). These improvements included the Executive Function (Working Memory) and Attention-Speed (Attention) for the AE group and the Attention-Speed (Attention and Speed) for the COMB group. Change in waist circumference in the AE group was found to mediate the intervention-related improvement in working memory (Path C’: *B* = 1.00, SE = 0.33, *p* = 0.003; 95% CI 0.35, 1.66; Path AB: *B* = − 0.25, SE = 0.14, 95% CI − 0.55, − 0.01). Mediation analyses showed no effect of the global FA and MD on the cognitive benefits for any group.

## Discussion

This paper presents the findings of the “Projecte Moviment” randomized control trial regarding the neuroprotective effect of AE, CCT and their combination (COMB) on cardiovascular risk factors and WM integrity outcomes in healthy physically inactive late-middle-aged adults.

In our study, participants in the AE group showed significant intervention-related changes in cardiovascular risk factors but not in WM integrity. Following a 12-week, 5-day-per-week, 45-min-per-day brisk walking program our participants in the AE group decreased in BMI, waist circumference and diastolic blood pressure. This finding is consistent with literature suggesting that PA improves metabolic profile and cardiovascular health (Bacon et al. 2004; Fagard 2001; Nystoriak & Bhatnagar 2018; Pinckard et al. 2019). It also underlines the benefits of a low-cost high-impact lifestyle intervention on healthy aging, which might lead to long-term molecular, structural, and functional long-term neuroprotective benefits (Stillman et al. 2016). In our previous cross-sectional studies, we reported a positive relationship between physical exercise and an inflammatory profile, brain volume, and cognition (Castells-Sánchez et al. 2020, 2021); however, we could not replicate the molecular and brain volume imaging findings after implementing the RCT (Castells-Sánchez et al. 2022). Similarly, although previous cross-sectional studies (Gow et al. 2012; Johnson et al. 2012; Liu et al. 2012) showed that higher PA levels are related to increased global FA and local FA levels in the corpus callosum, superior longitudinal fasciculus and arcuate fasciculus, several RCT failed to find significant changes on WM microstructure (Clarck et al. 2019; Sexton et al. 2020; Voss et al. 2013). Therefore, it seems that longer interventions are needed to detect greater changes in brain WM microstructure. In fact, the parameters of the activity, specifically duration, may be a critical aspect of an exercise intervention. It is also indicated (Lin et al. 2015; Stillman et al. 2020) that exercise neuroprotective effects might be modified by health status, sex and age, highlighting that people with significant cardiovascular risk factors (type 2 diabetes, metabolic syndrome, etc.), with less than 50 years, and men showed more benefits. This may also explain the absence of changes in lipid profile and WM integrity in our healthy late-middle-aged sample overrepresenting women (62%) unlike other trials with clinical population (overweight, obese or metabolic syndrome) (Cho et al. 2011; Erickson et al. 2022; Kraus et al. 2002; Pettman et al. 2009).

Participants in the CCT group exhibited a significant change in the global MD compared to the waitlist control group. Although the intragroup differences between baseline and follow-up were not statistically significant, there was a trend towards decreased MD in the CCT group and a slight increase in MD in the waitlist control group. These findings align with existing literature (Cao et al. 2016; de Lange et al. 2016; Engvig et al. 2012), which suggests that CCT may maintain, rather than alter WM integrity, highlighting its potential positive role in preserving WM microstructure. Surprisingly, the participants in the CCT group also showed a significant reduction in triglycerides and blood pressure. One potential explanation for these changes is that taking part in a lifestyle behavior project could have motivated participants to make additional modifications in their daily routines. In future studies, it may be worthwhile to explore the impact of diet explicitly on these outcomes. Moreover, it is also interesting to investigate how cardiovascular status at baseline can influence the effectiveness of these interventions.

As a significant finding of our study, we observed that the combined AE and multimodal CCT (COMB intervention) led to improvements in anthropometric measures, including BMI and diastolic blood pressure, as well as WM integrity, with increased local FA and decreased local MD, in comparison to the control group. These findings are particularly valuable given the scarcity of such studies. Notably, our results reinforce previous research, which also reported decreased local MD in the temporal lobe (Lövdén et al. 2012), frontal lobe, and subcortical areas (Takeuchi et al. 2020), supporting the benefits of our combined training approach in preserving brain structures and cognitive functions, such as memory, attention-speed, and executive function (Anderson-Hanley et al. 2012; Fabre et al. 2002; Roig-Coll et al. 2020). It is worth highlighting that the cardiovascular benefits observed in the COMB group were similar to those seen in the AE group, but only the COMB group showed further (local) improvement in WM integrity. Additionally, the lack of significant improvements in local WM integrity after single CCT suggests that the physiological benefits derived from AE may enhance the effect of CCT on WM. These findings underscore the potential additive effects of combining AE and CCT, highlighting the value of our COMB intervention for both cardiovascular health and brain structure.

Our study found that changes in PA and CRF for the AE and COMB groups (as previously reported in Roig-Coll et al. 2020) were not related to changes in the cardiovascular risk factors and the global and local diffusivity parameters. Findings from Voss et al., (2013), which reported a significant correlation between an increase in CRF and an increase in FA following a 1-year AE program, suggest that a minimum intervention duration may be a key parameter of the program for physical exercise intervention-related benefits.

We found no significant moderating effect of age on the changes in cardiovascular risk factors and WM integrity. This lack of effect could be attributed to our participants' relatively young and limited age range, as brain health tends to decline more in older and clinical populations, potentially showing a larger intervention effect in those groups (Erickson et al. 2014). However, we did observe significant sex differences in moderating intervention-related changes. Women showed greater reductions in waist circumference and diastolic pressure following AE, while men exhibited a more significant reduction in LDL levels. It's important to interpret moderation results for the CCT and COMB groups with caution due to potential biases in sex representation in those groups. Nonetheless, our findings are consistent with existing literature suggesting that sex plays a relevant role as a moderator of AE's neuroprotective effects. This is likely due to sex-specific adaptations in the respiratory, musculoskeletal, and cardiovascular systems, as well as the influence of sex hormones on these physiological processes (Barha and Liu-Ambrose 2018; Barha et al. 2019; Castells-Sánchez et al. 2020, 2021, 2022).

To provide further evidence regarding the mechanisms underlying the cognitive benefits observed in AE (Executive and Attention-Speed) and in COMB groups (attention-speed) (Roig-Coll et al. 2020) in this sample, we investigated the potential mediating effects of changes in the cardiovascular risk factors and the global FA and MD. We found that the relationship between AE and working memory was mediated by the waist circumference. Specifically, AE improved working memory but waist circumference as a mediator diminished the benefit. Further research is needed to understand better the biological mechanisms involved in the AE-related cognitive benefits.

## Limitations

Our results may be influenced by methodological factors, such as the brief duration of the intervention and the small sample size reducing the power to detect mediation effects (Stillman et al. 2016). Future studies should use larger samples and ensuring unbiased representation of age and sex. Moreover, they should examine how diet patterns influence cardiovascular risk factors and anthropometric and blood sample measures.

We must also acknowledge potential ceiling effects in our study. For example, larger variance in cardiovascular health at baseline would allow to assess its effect on AE, CCT and COMB benefits as well as its relationship with changes in WM integrity.

Finally, we employed a single-acquisition of DTI scans to reduce participant fatigue and maintain compliance; however, such approach does not allow more advanced corrections for susceptibility-related distortions.

## Conclusion

In conclusion, this study provides evidence of the clinical relevance of lifestyle interventions, such as AE and CCT, and the potential additive advantages of combining them. The AE program was successful in improving anthropometric measures related to cardiovascular health, while the CCT was partially successful in maintaining WM integrity. However, when AE and CCT were applied in combination (COMB), the AE-related benefits could boost the CCT’s neuroprotective effect leading to improved local WM integrity in frontal and temporal structures, usually affected in pathological aging. This strongly suggests that COMB intervention could have a strong effect on the WM microstructure even in short interventions. Finally, our study further elaborated on how individual characteristics, such as sex may impact intervention-related benefits, thus emphasizing more personalized approaches.

## Data Availability

Data will be available upon reasonable request.
